# A hybrid model for refining gross primary productivity estimation by integrating multiple environmental factors

**DOI:** 10.1016/j.mex.2024.103091

**Published:** 2024-12-09

**Authors:** Zhilong Li, Ziti Jiao, Zheyou Tan, Chenxia Wang, Jing Guo, Sizhe Chen, Ge Gao, Fangwen Yang, Xin Dong

**Affiliations:** a State Key Laboratory of Remote Sensing Science, Beijing Normal University, Beijing 100875, China; b Institute of Remote Sensing Science and Engineering, Faculty of Geographical Science, Beijing Normal University, Beijing 100875, China; c Beijing Engineering Research Center for Global Land Remote Sensing Products, Beijing Normal University, Beijing 100875, China

**Keywords:** Random forest, Carbon cycle, Two-leaf light use efficiency model, Stress variables, Large-scale GPP estimation via combining RF technique with TL-LUE model

## Abstract

Environmental factors lead mainly to the uncertainty of gross primary productivity estimation in most light use efficiency (LUE, ε) models since the simple physical formulas are inadequate to fully express the overall constraint of diverse environmental factors on the maximum ε (ε_max_). In contrast, machine learning has the natural potential to detect intricate patterns and relationships among various environmental variables. Here, we presented a hybrid model (TL-CRF) that utilizes the random forest (RF) technique to incorporate various ecological stress factors into the two-leaf LUE (TL-LUE) model, meanwhile, seasonal differences in the clumping index (CI) on a global scale are considered to adjust seasonal patterns of canopy structure. The comprehensive integration of complex environmental variables based on this RF submodule is conducive to scaling theoretical ε_max_ to actual ε as much as possible. The proposed TL-CRF model considerably improves global GPP estimation by complementing innate advantages between the process-based and data-driven models.

• The seasonal CI averages in different stages of the leaf life cycle are estimated for different vegetation types on a global scale.
• Various environmental stress factors are integrated via the RF technique.
• The RF submodule is embedded into the TL-LUE model to establish a hybrid model.

## Graphical abstract

**Figure:**
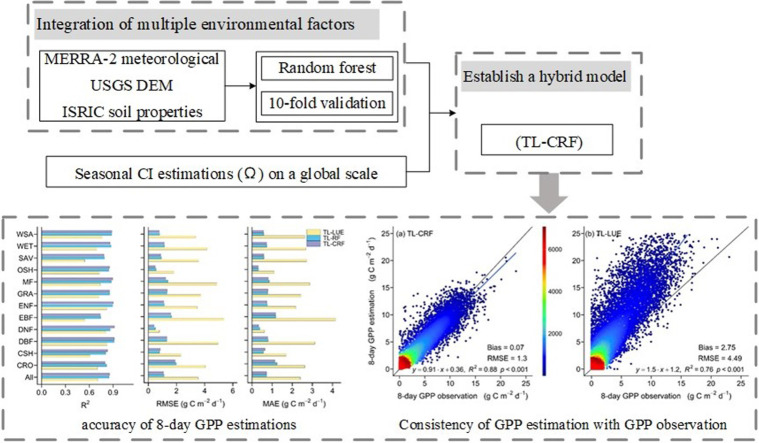

Specifications table

**Table:** 

Subject area:	Environmental Science
More specific subject area:	Terrestrial productivity estimation
Name of your method:	Large-scale GPP estimation via combining RF technique with TL-LUE model
Name and reference of original method:	Two-leaf light use efficiency model [1] Random Forest algorithm [2]
Resource availability:	FLUXNET2015 dataset: https://fluxnet.org/data/download-data/ AmerFlux network: https://ameriflux.lbl.gov/data/download-data/ NASA MERRA-2: https://disc.gsfc.nasa.gov/datasets?page=1 MODIS LP DACC: https://lpdaac.usgs.gov/product_search/?status=Operational ISRIC SOIL GRIDS: https://www.soilgrids.org/ USGS: https://earthexplorer.usgs.gov/.

## Background

Gross primary productivity (GPP) is the total amount of CO_2_ uptake by photosynthesis within a unit of time [3], which has been a key indicator for assessing the carbon cycle of terrestrial ecosystems and quantifying the feedback of vegetation to climate change [4]. Recently, the light use efficiency (LUE, ε) models on the basis of the LUE theory have made great progress, which has been widely used for simulating global or regional GPP due to clear principle, concise framework, and low-cost running. Among them, the two-leaf light use efficiency (TL-LUE) model further improves GPP estimations by separating the whole canopy into sunlit and shaded leaves relying on the constant clumping index (CI) estimation (Ω). With the advancement of long-term global CI products, It is possible to integrate the temporal variation of CI into the study of remote sensing and land surface simulation [5]. The main uncertainty of GPP estimations in the LUE models originates from complex environmental factors such as water content, temperature, and solar radiation. The actual ε is generally limited by various environmental stress factors, and reaches the theoretical maximum ε (ε_max_) only under ideal environmental conditions with adequate nutrients and appropriate water and heat content. At present, most LUE models have considered only two to four environmental factors via the multiplication principle or the law of the minimum, which is insufficient for quantifying the synthesis scale of complex environmental factors on the ε_max_ (σ). With the advancement of artificial intelligence techniques, machine learning (ML) can learn some varying parameters of a model from rich datasets [6], which likely leads to a breakthrough for integrating various environmental factors. The previous study highlighted the advantages of ML in terms of optimizing parameters [7]. The goal of the hybrid model was to replace the uncertainty factors and processes with machine learning techniques to effectively access information from earth observation data while preserving physical consistency and interpretability [8]. The paper set out to refine global GPP estimations by integrating various environmental stress factors via the random forest (RF) technique. Apart from the latest paper, this work offers a fresh perspective on constructing a hybrid model.

## Method details

**Fig. 1:**
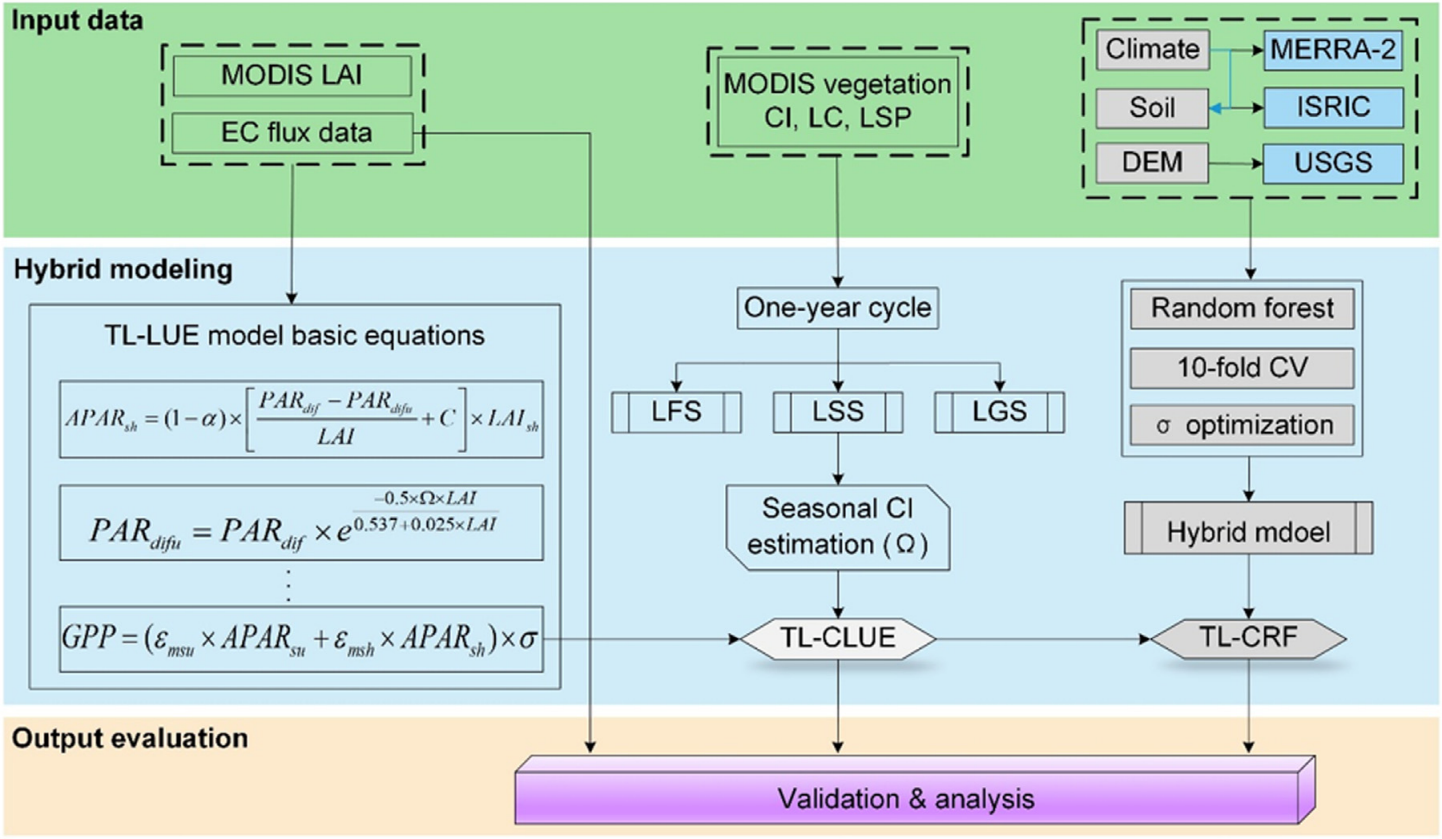
Workflow chart of improvement of GPP estimation in the TL-CRF model by integrating multiple environmental stress factors using the RF model while accounting for seasonal differences in CI in different leaf growth seasons (LFS, LSS, and LGS). The study updates the TL-CLUE model to a global scale. Furthermore, the σ estimation is improved by integrating various environmental stress factors including MERRA-2 meteorological factors (i.e. water content, temperature, and wind speed), ISRIC soil properties (i.e. soil silt, clay, and organic carbon), and USGS elevation (Elev). The TL-CRF model was trained and validated based on global eddy covariance flux data from the FLUXNET2015 dataset and the AmeriFlux network.

As shown in Fig. 1, the paper aimed to improve global GPP estimation by combining the TL-LUE model with the RF submodule for integrating diverse environmental stress factors after considering seasonal differences in CI. Canopy spatial structure can be described by CI [9], of which seasonal variations reflect changes in canopy structure [10,11]. Our latest work [12]showed an approximately 9.76 % improvement in GPP estimation across Northern America when the TL-LUE model adopts three seasonal CI averages (TL-CLUE). This paper attempted to update the TL-CLUE model to a global scale. Currently, some released remote sensing CI products with higher temporal resolution (eight-day and daily scale) remain a great uncertainty in their seasonal changes caused by short fluctuations in various environmental elements such as heavy rain and storms. Hence, we estimated three seasonal CI means for each vegetation type, which somewhat smooths uncertainty arising from the upriver steps of CI remote sensing retrieval by averaging. The one-year cycle of vegetation foliage life was divided into the leaf-scattering (LSS), leaf-gathering seasons (LGS), and leaf-off (LFS) according to the MODIS land surface phenology (LSP, MCD12Q2 V061) data deriving from the two-band Enhanced Vegetation Index with no blue band (EVI2). The existing knowledge argues that canopy pigments reflected in EVI2 are strongly associated with canopy structures depicted in CI, and thus this MODIS LSP data is reasonable for seasonal division.

Furthermore, the RF model is used to refine the σ estimation accuracy since the machine learning algorithm has an obvious advantage in capturing the nonlinear relationships between the predictors and response [13]. The RF technique can identify the nonlinear interactions between variables by constructing multiple decision trees based on data and features [2]. The drivers of machine learning should be selected on the basis of the understanding of biophysical processes and data accessibility. Previous work reported that temperature, precipitation, solar radiation, and vegetation indices could influence the activity of vegetation photosynthesis [4]. Therefore, we used a comprehensive dataset of soil properties, meteorological factors, and topographic element as drivers of photosynthesis, and different degrees of each of these factors are involved in the photosynthesis process. For example, terrestrial water storage (TWS) is essential for chemical reactions of photosynthetic processes. The RF model was utilized to develop a σ procedure comprehensively encoding the functional relationships between various environmental factors and ε_max_ in diverse terrestrial ecosystems.

Finally, a hybrid model (TL-CRF) was established by embedding the above RF submodule into the TL-CLUE model. The hybrid model is helpful in leveraging natural complementary advantages between “rationalism” in the mechanistic model and “empiricism” in the data-driven model [14]. The TL-CRF model not only utilizes the flexibility of the RF technique to learn control of various environmental conditions on photosynthetic potential from plentiful earth observation data but also has strong mechanistic interpretability due to retaining the host architecture of the TL-LUE model. The σ is predicted by the RF submodule driven under various environmental factors including meteorological variables, soil properties, and elevation, then the σ values are transferred to the TL-CLUE host module to estimate GPP.

### TL-LUE model

Compared with the MOD17 GPP algorithm, the TL-LUE model further improves GPP estimations by separating the vegetation canopy into shaded and sunlit leaves on the basis of CI. The fundamental theory is as follows:

(1) $$GPP=f\left(PAR,LAI,\theta ,\beta ,\alpha ,\Omega ,\varepsilon_{msu},\varepsilon_{msh}\right)\times \sigma$$

GPP can be specifically estimated as follows:

(2) $$GPP=\left(\varepsilon_{msu}\times APAR_{su}+\varepsilon_{msh}\times APAR_{sh}\right)\times \sigma$$

where *ε_msu_* and *APAR_su_* are the *ε_max_* and canopy absorbed photosynthetically active radiation (*APAR,* MJ m^–2^ d^–1^) values of sunlit leaves, respectively; *ε_msh_* and *APAR_sh_* are the *ε_max_* and *APAR* of shaded leaves, respectively; and *σ* represents the scale of the environmental stress factors on *ε_max_*, ranging from 0 to 1. The calculations of *σ, APAR_su,_* and *APAR_sh_* are as follows:

(3) $$\sigma =h\left(T_{a\min}\right)\times g\left(VPD\right)$$

(4) $$APAR_{sh}=\left(1-\alpha \right)\times \left[\frac{PAR_{dif}-PAR_{difu}}{LAI}+C\right]\times LAI_{sh}$$

(5) $$APAR_{su}=\left(1-\alpha \right)\times \left[PAR_{dir}\times \frac{\cos(\beta )}{\cos(\theta )}+\frac{PAR_{dif}-PAR_{difu}}{LAI}+C\right]\times LAI_{su}$$

where *VPD* and *T_amin_* are daily vapor pressure deficit (hPa) and minimum air temperature, respectively; *PAR_dir_* and *PAR_dif_* are the direct, and diffuse partition of PAR, respectively; *PAR_difu_* is the diffuse PAR under the canopy [15]; *α* is the canopy albedo depending on vegetation types (Table 1), *θ* is the solar zenith angle; *β* represents the sun-leaf angle in the spherical canopy, which is usually taken to 60°; *C* denotes the multiple scattering effects of direct radiation inside the canopy; *LAI_su_* and *LAI_sh_* are *LAI* of sunlit and shaded leaves, respectively [15]. These variables can be calculated as follows:

(6) $$PAR_{difu}=PAR_{dif}\times e^{\frac{-0.5\times \Omega \times LAI}{0.537+0.025\times LAI}}$$

(7) $$PAR_{dir}=PAR-PAR_{dif}$$

(8) $$PAR_{dif}=PAR\times \left(0.7527+3.8453\times R-16.316\times R^{2}+18.962\times R^{3}-7.0802\times R^{4}\right)$$

(9) $$R=\frac{PAR}{0.5\times \cos\left(\theta \right)\times S_{0}}$$

(10) $$LAI_{\text{su}}=2\times \cos\left(\theta \right)\times \left(1-e^{\frac{-0.5\times \Omega \times LAI}{\cos(\theta )}}\right)$$

(11) $$LAI_{\text{sh}}=LAI-LAI_{\text{su}}$$

where Ω means the foliage CI estimation (Table 1), plays an indispensable role in the distinction of solar radiations and separation of the canopy; and *S_0_* is the solar constant generally set to 1367 w *m*^−2^.

**Table 1:** Parameters of each vegetation type required by the TL-LUE model.

**Vegetation**^1^	**CRO**	**CSH**	**DBF**	**DNF**	**EBF**	**ENF**	**GRA**	**MF**	**OSH**	**SAV**	**WET**	**WSA**
ε_msh_ (g C MJ^–^1)^2^	4.01	2.10	3.44	1.89	2.83	2.99	3.80	2.93	2.73	3.28	2.90	2.86
ε_msu_ (g C MJ^–^1)^2^	1.34	0.70	0.85	0.70	1.08	0.89	1.12	1.02	0.67	2.41	1.19	1.79
T_amin_min_ ( °C)	−5.67	−6.33	−5.00	−5.67	−4.50	−5.67	−5.00	−5.67	−6.33	−5.67	−4.00	−5.6
T_amin_max_ ( °C)	12.02	8.71	8.94	9.38	9.09	8.31	12.02	9.00	8.80	10.00	8.61	11.39
VPD_max_ (hPa)	41.67	44.00	32.83	32.00	37.67	42.67	45.00	35.33	43.33	37.67	41.00	38
VPD_min_ (hPa)	8.37	7.90	8.37	7.90	8.37	8.37	8.37	8.37	8.37	8.37	9.30	8.37
α^3^	0.20	0.18	0.16	0.13	0.17	0.13	0.21	0.15	0.19	0.19	0.19	0.20
Ω^4^	0.90	0.80	0.80	0.60	0.80	0.60	0.90	0.70	0.80	0.80	0.87	0.80

1 CRO: cropland; CSH: close shrub; DBF: deciduous broadleaf forest; DNF: deciduous needleleaf forest; EBF: evergreen broadleaf forest; ENF: evergreen needleleaf forest; GRA: grassland; MF: mixed forest; OSH: open shrub; SAV: savannas; WET: wetland; WSA: woody savannas.

2 [16], the _Ɛmax_ of shaded and sunlit leaves was estimated on the basis of global flux site data.

3 [16,17], the α is the canopy albedo.

4 [18], the Ω is the vegetation clumping index estimation, which plays a vital role in dividing the canopy into the sunlit and shaded leaves.

The *h(T_amin_)* and *g(VPD)* in Eq. (3) can be specifically expressed as follows:

(12) $$h\left(T_{a\min}\right)=\left\{\begin{array}{c} 0\begin{array}{cc} \begin{array}{cc} & \begin{array}{cccc} & & & T_{a\min}\leq T_{a\min\_\min} \end{array} \end{array} & \end{array}\\ \frac{T_{a\min}-T_{a\min\_\min}}{T_{a\min\_\max}-T_{a\min\_\min}}\begin{array}{cc} & T_{a\min\_\min}<T_{a\min}<T_{a\min\_\max} \end{array}\\ 1\begin{array}{cc} \begin{array}{cc} & T_{a\min}\geq T_{a\min\_\max} \end{array} & \end{array} \end{array}\;\;\;\;\;\;\;\;\;\;\;\;\;\;\right\}$$

(13) $$g\left(VPD\right)=\left\{\begin{array}{cc} 0 & VPD_{\max}\leq VPD\\ \frac{VPD_{\max}-VPD}{VPD_{\max}-VPD_{\min}} & VPD_{\min}<VPD<VPD_{\max}\\ 1 & VPD_{\min}\geq VPD \end{array}\right\}$$

where *T_amin__*_min_ and *VPD_min_* are daily T_amin_ and VPD when photosynthesis decreases to 0, respectively; *T_amin__*_max_ and *VPD_max_* are daily T_amin_ and VPD when photosynthesis reaches to the highest value (Table 1), respectively. These four parameters are closely related to vegetation types [16].

### Global CI estimations in different seasons of vegetation growing

Vegetation CI is one of the key parameters for describing the canopy structure of vegetation [9] and plays an essential role in ecological and meteorological modeling [19]. Previous studies [20,21] have argued that there are seasonal differences in CI, particularly for DBF and MF. With the development of global long-term CI datasets, it is feasible to incorporate seasonal changes into the land surface process simulations [5]. Our latest work [12] proposed the TL-CLUE model that considers the seasonal differences in CI into the TL-LUE model to characterize the seasonal differences in the canopy. As a result, the TL-CLUE model can reduce the GPP overestimations across the North America by approximately 9.76 % when employs three Ωs from three stages of leaf life instead of only constant Ω. Furthermore, this paper attempts to upgrade the TL-CLUE model to a global scale.

This paper divides the world into three altitudinal zones according to the seasonal characteristics of CI. Specifically, the northern hemisphere (NH) is the region north of 30°N excluding Greenland, and its total land area is approximately 106.34 million square kilometers; the southern hemisphere (SH) is the region south of −30°N excluding Antarctica, with the total land area of 6.583 million square kilometers; and the tropics (Trop) is between −30 °N and 30°N. Previous research has shown no distinct seasonal differences in CI in the Trop [20]. Therefore, the one-year cycle of leaf life is unnecessarily divided, and the optimal Ω is viewed as the annual average of CI in the Trop. For NH, as shown in Table 2, the one-year cycle of leaf growing is divided into the LSS, LGS, and LFS on the basis of four phenology thresholds including Greenup, MidGreenup, MidGreendown and Dormancy from the MODIS LSP dataset, which works effectively for tracking phenology of vegetation [22]. In the NH, LGS is from MidGreenup to MidGreendown, LSS from Greenup to MidGreenup and from MidGreendown to Dormancy, and LFS before Greenup and after Dormancy. On the contrary, in the SH, its total land area is only approximately 6.2 % of NH, and the phenology features of vegetation are widely opposite to the NH. Therefore, in the SH, LFS is roughly from MidGreenup to MidGreendown; LSS from Greenup to MidGreenup and from MidGreendown to Dormancy; LGS before Greenup and after Dormancy. Additionally, the LGS and LSS can be combined into the leaf-on season (LOS).

**Table 2:** Four phenological thresholds are used to divide the one-year cycle of vegetation growing into different seasons in the Northern Hemisphere.

vegetation	Phenology threshold			
	Greenup	MidGreenup	MidGreendown	Dormancy
CRO	73	150	257	294
CSH	102	135	257	291
DBF	89	156	276	313
DNF	117	145	243	270
EBF	43	98	260	300
ENF	109	143	259	296
GRA	89	146	256	305
MF	89	146	257	281
OSH	97	156	249	288
SAV	81	152	192	249
WET	73	140	252	289
WSA	33	86	265	321

The seasonal Ωs from different seasons are key for the TL-CLUE model to capture the phase pattern of APAR. Moreover, the seasonal CI average is beneficial for mitigating the uncertainty caused by retrieval processes and upper-level remote sensing data (MODIS reflectance data). In the TL-CLUE model, the seasonal Ω_i_ is expected to optimize the description of fractions of solar radiation as follows:

(14) $$PAR_{difu}=PAR_{dif}\times e^{\frac{-0.5\times \Omega_{i}\times LAI}{0.537+0.025\times LAI}}$$

where Ω_i_ is a CI estimation in the i^th^ season including leaf-off, leaf-scattering, and leaf-gathering seasons.

To estimate the Ω value of the corresponding season for global vegetation types, the paper selected the MODIS CI product inversed from the MODIS bidirectional reflectance distribution function (BRDF) on the basis of the hotspot-adjusted model and a backup algorithm [23], which remarkably enhanced the data quality. Finally, the corresponding seasonal Ωs for different vegetation types in three latitude regions (Table 3) are determined by synthesizing the MODIS CI and the published literature related to the CI [10,20,21]. The vegetation in the SH can equally adopt the same Ω values as those in the NH, with the exception that the vegetation phenology in the SH is fundamentally opposite to that in the NH.

**Table 3:** Seasonal Ω values for different vegetation types in the Northern and Southern Hemisphere, and Tropics. The annual Ω is estimated for the different vegetation types in the Tropics due to less seasonal differences in CI.

Region	Vegetation	Ω			
		LFS	LOS	LSS	LGS
NH, SH	CRO	0.86±0.05	0.72±0.07	0.74±0.07	0.70±0.08
	CSH	0.75±0.07	0.67±0.08	0.70±0.08	0.65±0.07
	DBF	0.74±0.06	0.66±0.09	0.69±0.08	0.64±0.10
	DNF	0.71±0.05	0.59±0.05	0.61±0.04	0.57±0.05
	EBF	0.73±0.04	0.62±0.05	0.65±0.05	0.59±0.05
	ENF	0.63±0.08	0.53±0.09	0.55±0.08	0.51±0.09
	GRA	0.82±0.07	0.69±0.08	0.72±0.08	0.67±0.07
	MF	0.72±0.03	0.65±0.09	0.63±0.09	0.59±0.09
	OSH	0.78±0.07	0.68±0.07	0.7 ± 0.07	0.65±0.06
	SAV	0.83±0.05	0.68±0.06	0.70±0.06	0.66±0.06
	WET	0.83±0.08	0.72±0.08	0.75±0.08	0.69±0.08
	WSA	0.84±0.08	0.69±0.08	0.71±0.07	0.66±0.08
Trop	CSH	0.77±0.04			
	DBF	0.68±0.06			
	DNF	0.58±0.03			
	EBF	0.69±0.04			
	ENF	0.51±0.05			
	GRA	0.78±0.05			
	SAV	0.75±0.05			
	WET	0.78±0.08			
	WSA	0.73±0.05			
	CSH	0.77±0.04			

### Integration of various environmental stress factors via RF

ML has a great advantage in identifying nonlinear patterns and interactions in multiple variable datasets [24], which provides a way to integrate various environmental stress factors in LUE models. RF algorithm [25,26] blends the Bagging integration algorithm and CART decision tree construction. It randomly selects node features on the basis of the parallel combination of decision trees. The best model is determined by the voting system, which is a complex and strong classifier [27] made up of numerous weak classifiers (decision trees).

The _Ɛmax_ is limited by numerous environmental factors such as meteorological variables, hydrological elements, and nutrients. However, the TL-LUE model only selects VPD and daily the minimum air temperature (T_amin_) to constrain the _Ɛmax_, which most likely leads to an error in GPP estimations. The previous study has argued climate variables are crucial for global GPP projection [28]. Moreover, there are wide nonlinear relationships between various environmental factors and GPP [29]. To improve the accuracy of σ estimation, this paper attempts to integrate various environmental factors using RF technique, including meteorology, vegetation, soil properties, and topography. In theory, the real σ value can be indirectly derived by the TL-CLUE model with considering seasonal differences in CI. As shown in Eq. (15), the RF model is established to express the quantitative relationship between the σ and multiple environmental factors including terrestrial water storage (TWS), T_amin_, VPD, soil temperature (T_s_), maximum air temperature (T_amax_), total cloud area fraction (FCA), wind speed (S_w_), soil silt (S_t_), soil nitrogen (S_n_), soil bulk density (S_d_), soil clay (S_y_), SOC (soil organic carbon), Elev (elevation), and P (precipitation).

(15) $$\sigma =r\left(TWS,T_{s},VPD,T_{amin},T_{amax},S_{w},P,FCA,Elev,S_{t},S_{n},S_{d},S_{y},SOC\right)$$

The training process of the RF model can be carried out in R platform. In addition, we also compared the RF model with other ML models like support vector machine (SVM), gradient boosted regression tree (GBRT), and artificial neural networks (ANN). On the basis of random 70 % of global site observed data, these ML models for different vegetation types were trained and evaluated by tenfold cross-validation via coefficient of determination (R^2^), root mean square error (RMSE), and mean absolute error (MAE). Finally, the RF model was chosen for this paper since its performance outperforms the other ML models.

### Design a hybrid model to improve GPP estimations

Recently, the concept of a hybrid model has been proposed, which involves combining process models and data-driven models into a single end-to-end modeling system [30], which is beneficial for improving the earth system or components like sea surface temperature [31]. Here, an initial objective of this paper was to improve the accuracy of GPP estimations (Eq. (16)) via embedding the RF submodule into the TL-CLUE model (TL-CRF). The TL-CRF model can utilize the flexibility of the RF technique to recognize the nonlinear relationships among diverse environmental factors.

(16) $$GPP=f\left(PAR,LAI,\theta ,\beta ,\alpha ,\Omega_{i},\varepsilon_{msu},\varepsilon_{msh}\right)\times r\left(TWS,T_{s},\cdots ,SOC\right)$$

In addition, to investigate the effect of seasonal differences in CI on integrating multiple environmental factors via ML technique, global GPP is also estimated by Eq. (17) via only combining the RF submodule into the TL-LUE model without considering seasonal Ω values (TL-RF). The only difference between the TL-RF and TL-CRF models is that TL-RF still uses the endless Ω for each vegetation type but the TL-CRF model adopts three seasonal Ωs from different stages of leaf growing on a global scale.

(17) $$GPP=f\left(PAR,LAI,\theta ,\beta ,\alpha ,\Omega ,\varepsilon_{msu},\varepsilon_{msh}\right)\times r\left(TWS,T_{amin},\cdots ,VPD\right)$$

### Model evaluation and statistics analysis

The accuracy of GPP estimation was evaluated against a test dataset (the remaining 30 % of global site flux data). The performances of the TL-CRF, TL-RF, and TL-LUE models for different vegetation types were assessed by using R^2^ (Eq. (18)), RMSE (Eq. (19)), and MAE (Eq. (20)). Furthermore, consistency between the GPP estimations and the GPP observations was investigated by linear regression.

(18) $$R^{2}=\frac{\sum_{i=1}^{n}{\left(v_{mi}-\bar{v_{o}}\right)}^{2}}{\sum_{i=1}^{n}{\left(v_{oi}-\bar{v_{o}}\right)}^{2}}$$

(19) $$RMSE=\sqrt{\frac{\sum_{i=1}^{n}{\left(v_{oi}-v_{mi}\right)}^{2}}{n}}$$

(20) $$MAE=\frac{\sum_{i=1}^{n}|v_{oi}-v_{mi}|}{n}$$

where v_o_ and v_m_ are observed and simulated values, respectively, *i* represents the ordinal value, and n is the size of the resampled point.

### Method validation

**Fig. 2:**
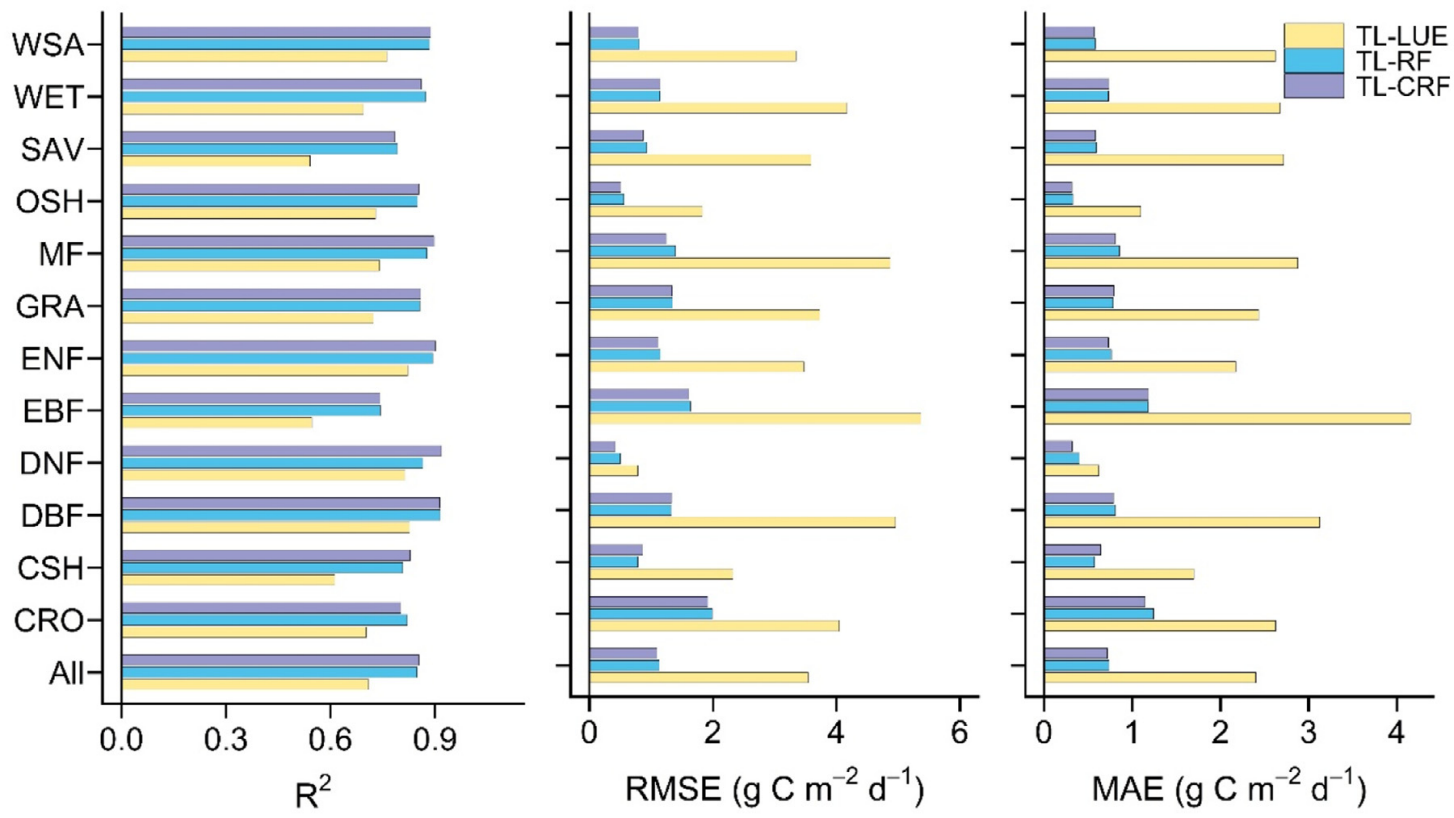
The accuracy of eight-day GPP estimations in the TL-LUE (yellow), TL-RF (blue), and TL-CRF modes (purple), and the uncertainty of the GPP estimations is measured by the RMSE (g C m^–2^ d^–1^) and MAE (g C m^–2^ d^–1^).

As shown in Fig. 2, the TL-RF model remarkably improves the GPP estimations with higher R^2^ and lower RMSE and MAE, as opposed to the TL-LUE model. Compared the TL-LUE model, R^2^ values in the TL-RF model are increased by approximately 0.05∼0.09 for DNF, ENF, and DBF; 0.118∼0.138 for CRO, OSH, WSA, GRA, and MF; 0.18∼0.25 for WET, CSH, EBF, and SAV (Fig. 2). Both the RMSE and MAE in the TL-RF model are lower approximately 70∼76 % for WSA, SAV, DBF, WET, MF, EBF, and OSH; 64∼65 % for EBF, CSH; 36∼51 % for CRO and DNF than those in the TL-LUE model (Fig. 2). These results indicate that the uncertainty of GPP estimation resulting from environmental factors would be largely reduced by the RF technique. Moreover, compared with the TL-RF model, the GPP estimation is slightly improved by the TL-CRF model considering the seasonal difference in CI (Fig. 2). The R^2^ of the TL-CRF model is hardly equal to the TL-RF model for most vegetation types (Fig. 2). The uncertainties of global GPP estimations in the TL-CRF model (Fig. 2) are only approximately 3 % (RMSE) lower than those in the TL-RF model without seasonal differences in CI, which may be linked to CI values ranging from 0 to 1. Additionally, the potential information on seasonal CI likely does not play a remarkable role in the integration process of the seasonal variation of multiple environmental factors based on the RF algorithm. In terms of overall vegetation types (Fig. 2), the performance of the TL-CRF model (R^2^=0.85, RMSE=1.1 g C m^–2^ d^–1^, MAE=0.72 g C m^–2^ d^–1^) is higher than that of the TL-LUE model (R^2^=0.71, RMSE=3.54 g C m^–2^ d^–1^, MAE=2.40 g C m^–2^ d^–1^).

**Fig. 3:**
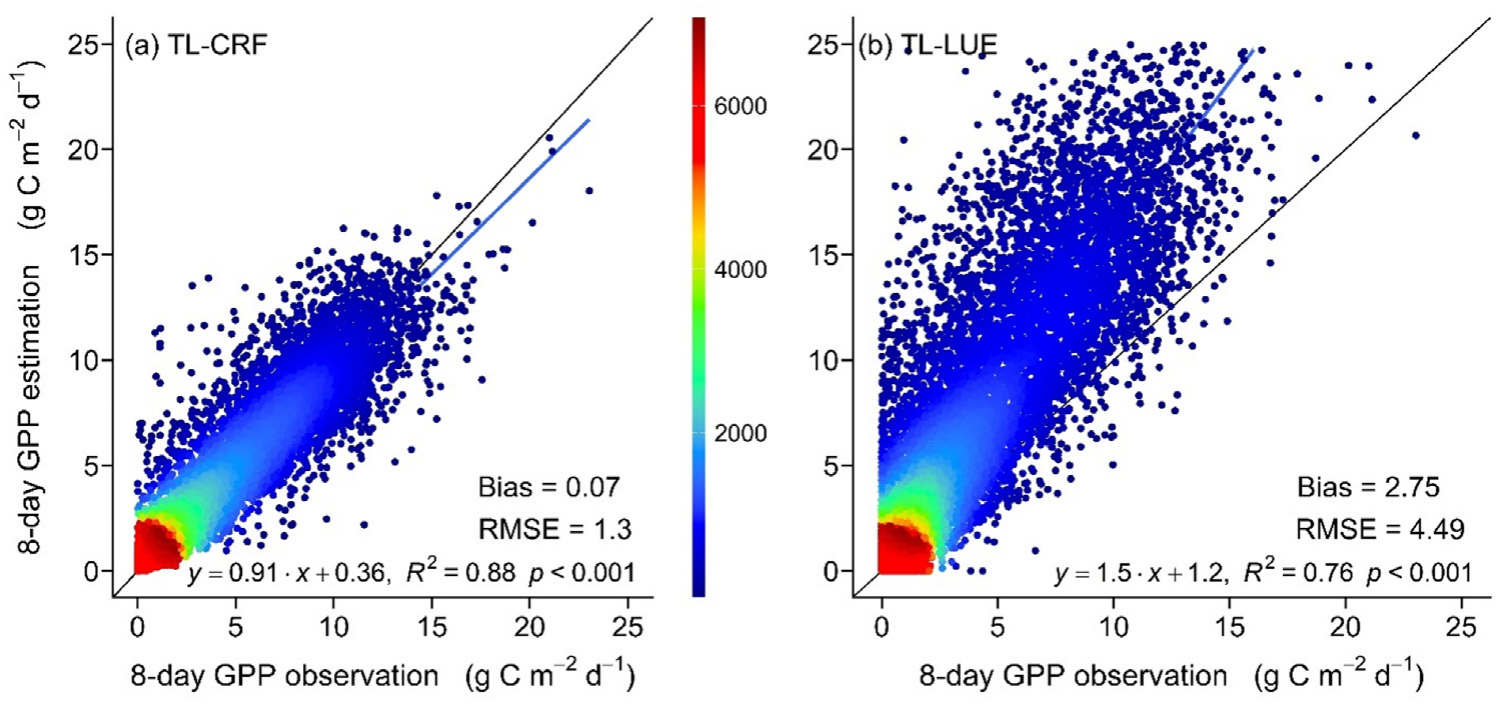
Lineal regression between the observed GPP and the GPP estimated by the TL-CRF(a), and TL-LUE(b) models, and the black solid line is a 1:1 theoretical line. The uncertainty of GPP estimations is measured by the RMSE (g C m^–2^ d^–1^).

As can be seen from Fig. 3, the regression line in the TL-CRF model is closer to the 1:1 theoretical line than that in the TL-LUE model, revealing that GPP estimations in the TL-CRF model are more consistent with the GPP observations (Fig. 3). The R^2^ (0.88) of the TL-CRF model is higher than that (0.76) of the TL-LUE model (Fig. 3). Additionally, compared with the TL-LUE model, the RMSE and Bias are reduced by approximately 71 and 97 % in the RF-LUE model, respectively (Fig. 3), revealing that the TL-CRF model can effectively reduce the uncertainty of GPP estimations.

**Fig. 4:**
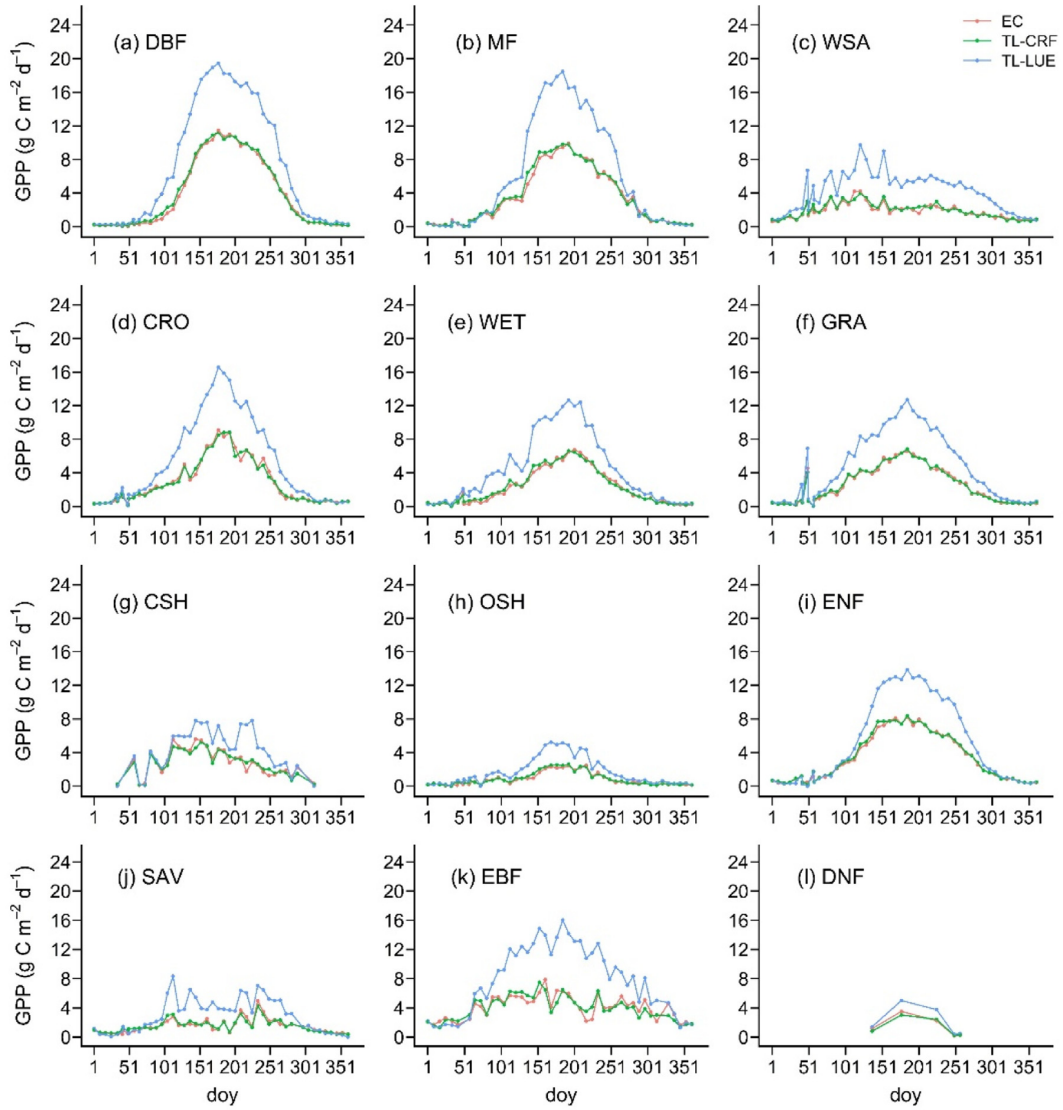
Seasonal changes of eight-day GPP in different vegetation types over 2002∼2020 in the Northern Hemisphere (north of 30°N). The red line is the observed GPP at EC (eddy covariance) flux sites, and the green and blue lines represent GPP estimated by the TL-CRF and TL-LUE models, respectively.

The temporal characteristics of GPP estimated by the TL-CRF model are in line with those of the site-observed GPP (Fig. 4). On the contrary, there are large fluctuations and differences in GPP between the TL-LUE model and the site observation, although the seasonal trend of GPP estimations in the TL-LUE model roughly corresponds to the site GPP observations. In the leaf-on season, the TL-LUE model visibly overestimates GPP for different vegetation types (Fig. 4), particularly at the growth peak. The TL-CRF model significantly decreases this GPP overestimation for most vegetation types by integrating various environmental stress factors, as shown in Fig. 4. In the leaf-off season, both GPP estimations in the TL-RF and TL-LUE models are nearly close to the GPP observations around 0 (Fig. 4). GPP increases from the end of the leaf-off season till the maximum GPP occurs at the peak growing. Although both the TL-LUE and TL-CRF models are in accord with the observed GPP in terms of general trends, the magnitude of GPP estimations in the TL-CRF is significantly equal to that of the GPP observations.

## Limitations

Although multiple environmental factors were integrated via RF technique, some incidental land surface processes were not incorporated into the model due to a lack of clear interpretation of their mechanism and difficulty in data accessibility. A large loss of carbon from ecosystems in a short period is generally triggered by extreme events like wildfires, high air temperatures, abrupt permafrost thaw, heatwaves, floods, tornadoes, and sandstorms. The attribution of these extreme events resulting from human interference in climate poses a significant issue to Earth system science [32]. Additionally, this paper did not distinguish the differences in vegetation photosynthetic rate between C3 and C4 plants. Currently, the MODIS yearly land cover product used in this paper has not yet further divided vegetation into C3 and C4 plants, as two fundamental plant functional types. Moreover, the performance of the RF model is partially limited by the quality of flux data and uneven site global distribution. Additionally, Uncertainty in GPP estimations is probably caused by the imperfect matching of spatial information among various driving variables from different data resources. Future research is needed to develop a reliable data fusion algorithm for complete surface observation data.

## Ethics statements

The work does not involve any ethical issues.

## CRediT authorship contribution statement

**Zhilong Li:** Conceptualization, Methodology, Writing – original draft. **Ziti Jiao:** Resources, Data curation, Project administration, Funding acquisition. **Zheyou Tan:** Writing – review & editing. **Chenxia Wang:** Investigation. **Jing Guo:** Formal analysis. **Sizhe Chen:** Visualization. **Ge Gao:** Supervision. **Fangwen Yang:** Software. **Xin Dong:** Validation.

## Declaration of competing interest

The authors declare that they have no known competing financial interests or personal relationships that could have appeared to influence the work reported in this paper.

## Data Availability

Data will be made available on request.
